# Identifying consistent allele frequency differences in studies of stratified populations

**DOI:** 10.1111/2041-210X.12810

**Published:** 2017-06-15

**Authors:** R. Axel W. Wiberg, Oscar E. Gaggiotti, Michael B. Morrissey, Michael G. Ritchie

**Affiliations:** ^1^ Centre for Biological Diversity Sir Harold Mitchell Building University of St Andrews St Andrews, Scotland United Kingdom; ^2^ Scottish Oceans Institute Gatty Marine Laboratory University of St Andrews East Sands St Andrews, Scotland United Kingdom

**Keywords:** allele frequency differences, CMH‐test, experimental evolution, pool‐seq, quasibinomial GLM, selection

## Abstract

1. With increasing application of pooled‐sequencing approaches to population genomics robust methods are needed to accurately quantify allele frequency differences between populations. Identifying consistent differences across stratified populations can allow us to detect genomic regions under selection and that differ between populations with different histories or attributes. Current popular statistical tests are easily implemented in widely available software tools which make them simple for researchers to apply. However, there are potential problems with the way such tests are used, which means that underlying assumptions about the data are frequently violated.
2. These problems are highlighted by simulation of simple but realistic population genetic models of neutral evolution and the performance of different tests are assessed. We present alternative tests (including Generalised Linear Models [GLMs] with quasibinomial error structure) with attractive properties for the analysis of allele frequency differences and re‐analyse a published dataset.
3. The simulations show that common statistical tests for consistent allele frequency differences perform poorly, with high false positive rates. Applying tests that do not confound heterogeneity and main effects significantly improves inference. Variation in sequencing coverage likely produces many false positives and re‐scaling allele frequencies to counts out of a common value or an effective sample size reduces this effect.
4. Many researchers are interested in identifying allele frequencies that vary consistently across replicates to identify loci underlying phenotypic responses to selection or natural variation in phenotypes. Popular methods that have been suggested for this task perform poorly in simulations. Overall, quasibinomial GLMs perform better and also have the attractive feature of allowing correction for multiple testing by standard procedures and are easily extended to other designs.

## 1 INTRODUCTION

With the increasing application of pooled genome sequencing (pool‐seq) approaches to population genomics (Boitard, Schlo, Nolte, Pandey, & Futschik, 2012; Ferretti, Ramos‐Onsins, & Pérez‐Enciso, 2013; Schlötterer, Kofler, Versace, Tobler, & Franssen, 2015; Schlötterer, Tobler, Kofler, & Nolte, 2014) researchers are interested in accurately quantifying allele frequency differences between populations and using these to infer the action of selection. Such data can provide us with insights into the evolutionary and demographic history of populations and to identify regions under selection and alleles that consistently differ in frequency between population substrata with different characteristics, across populations.

In particular, several tests of frequency differences have been used to compare allele frequencies at markers throughout the genome. The aim is usually to determine whether the frequencies of an allele at a particular marker (typically single nucleotide polymorphisms; SNPs) consistently differ between subsets of a population or whether such differences are consistent across replicated experimental evolution lines. This consistency is important because it provides a criterion to identify alleles that underlie the same trait in many populations and to distinguish consistent responses to selection from idiosyncratic responses or effects of drift in experimental evolution studies. A hypothetical example is where three replicate lines of a large mass selection treatment are set up from three separate but identical base line populations and allowed to evolve for several generations. Pooled whole genome sequencing (Pool‐seq; Schlötterer et al., 2014) can then be applied to determine the allele frequencies at different SNP markers throughout the genome. Markers that show a consistent difference across replicates are more likely to be functionally important in producing the phenotype under study.

Many of the statistical tests applicable to this kind of scenario are implemented in popular population genomic software tools (e.g. popoolation2) which make them routine to apply. However, here we find serious consequences of the misapplication of these tests that arise from two main sources. First, heterogeneity in allele frequency differences (e.g. arising from genetic drift) is often confused for significant main effects. Second, very little attention has been paid to pseudoreplication of allele counts that is inherent in pool‐seq experimental designs (where single chromosomes are “counted” multiple times). We show that these violations of statistical assumptions produce high false discovery rates (FDRs). These problems are highlighted by simulation and we present alternative tests for the analysis which improve inference.

### 1.1 The CMH‐test

The most widely used statistical method to compare allele frequencies is the Cochran‐Mantel‐Haenszel test (Cochran, 1954; Mantel & Haenszel, 1959)**,** an extension of Chi‐squared tests for multiple biological replicates. The CMH‐test considers 2 × 2 × *k* contingency tables. In the context of population genomics the rows and columns of each 2 × 2 table represent counts of different alleles (*X*) in different treatment lines or strata (*Y*) while *k* represents the number of biological replicates (e.g. different studies, populations, *Z*) (Agresti, 1996). In the CMH‐test the null hypothesis is that ‘*X* and *Y* are conditionally independent given *Z*’ (Agresti, 1996). A 2 × 2 table can be summarized by the conditional odds (O_*XYk*_) which measures the magnitude of the association between the factors *X* and *Y* at level *k*.

*If*

$$O_{XY1}=O_{XY2}=\cdots =O_{XYk},$$

then the odds ratios are homogeneous, the association between *X* and *Y* is the same at each level (*k*) of *Z*, and we are justified in describing the association with a single common odds ratio which can be tested for differences to 1 (Agresti, 1996). However, if the association between *X* and *Y* for the 2 × 2 tables is different across the *k* tables the test can give misleading results (Agresti, 1996; Landis, Heyman, & Koch, 1978; see also below). This assumption of homogeneity can be tested by, for example, the Woolf‐test (Woolf, 1955). Another assumption of the CMH‐test is that data contributing to each count within a cell of the contingency table are independent. The first assumption is frequently violated in real data. In fact, it is the pattern of consistency that is of interest. The second assumption is violated automatically in the design of pool‐seq experiments because allele counts obtained from reads directly are not independent draws from the treatment line or study population. Note also that this test assumes a pairing between the two treatment lines nested within replicates. Such a pairing may sometimes be biologically meaningful (e.g. if any two treatment and control lines were set up from the same source population). However, artificially pairing samples where no biological rationale exists is not ideal.

The CMH‐test as applied to genome‐wide marker data is implemented in the popular package popoolation2 (Kofler, Pandey, & Schlötterer, 2011), which aims to identify differences in allele frequencies that are consistent across biological replicates (Kofler et al., 2011). However, this package does not account for heterogeneity between replicates and thereby confuses this heterogeneity for a main effect. For example, Table 1 shows a hypothetical contingency table with an inconsistent allele frequency difference by any reasonable definition, for which the CMH‐test reports a significant result. This is surprising, because much of the rationale for using replicate lines in artificial evolution experiments is to distinguish genes that can be confidently identified as diverging due to selection rather than drift. Only the former should be consistent across lines. The genetic analysis tool PLINK (Purcell et al., 2007) also implements the CMH‐test and while the documentation recommends testing for heterogeneity, this is not routinely done in published studies. At the time of writing, the popoolation2 package had been cited 21 times with respect to the CMH‐test and PLINK's implementation of the CMH‐test 170 times in ‘Google Scholar’. While the popoolation2 package is never cited along with a test for heterogeneity, several of the studies citing PLINK also report tests for heterogeneity (e.g. Mero et al., 2010).

**Table 1:** A hypothetical set of contingency tables. The ‘A’ allele frequency difference between treatment lines ‘TL1’ and ‘TL2’ are not consistent across the three replicates. A CMH‐test gives the following significant results: Chi‐squared = 55.66, *df* = 1, *p* < .0001, common odds ratio = 6.98

Replicate	Treatment line	Allele	
		A	a
1	TL1	66	5
	TL2	90	3
2	TL1	72	3
	TL2	60	5
3	TL1	69	21
	TL2	6	72

### 1.2 Examples of the CMH‐test in the literature

Recently the CMH‐test has become highly popular in evolve and resequence (E&R) studies. Several such studies have considered data from a base population and three replicate treatment lines of *Drosophila melanogaster* sampled at various generations of experimental evolution under altered temperature regimes (Franssen, Nolte, Tobler, & Schlotterer, 2014; Kapun, Van Schalkwyk, McAllister, Flatt, & Schlötterer, 2014; Orozco‐terWengel et al., 2012a,2012b). Each generation, 500 females were sequenced by pool‐seq, and a CMH‐test was used to test if the differences in allele frequencies between treatments were consistent across replicates (Franssen et al., 2014; Orozco‐terWengel et al., 2012a,2012b). These studies identified regions indicative of haplotype blocks under selection by finding consistent, large average changes in allele frequencies across replicate treatment lines in response altered temperature regimes (Franssen et al., 2014). Another study based on the same experimental evolution dataset used three replicates of two selection regimes (Kapun et al., 2014). Single nucleotide polymorphism frequencies within inversions were used to infer changes in frequencies between the selection regimes. Consistency of inversion frequency differences across replicates was tested with the CMH‐test (Kapun et al., 2014). The study found significant, consistent changes between treatments across replicates, and the authors quantified the variation in changes of inversion frequencies. These studies do not report any attempts to test whether odds ratios across replicates are equal nor do they report how much allele frequency differences vary between replicates and they do not account for frequency variation that arises from coverage greatly exceeding the number of independent chromosomes in each pool, which is essentially pseudoreplication (Kolaczkowski, Kern, Holloway, & Begun, 2011).

Another E&R study considered adaptation to viral infection rates of the *Drosophila* C virus (DCV). Four replicates of three regimes were compared; ancestral, sham‐control and infected, where flies were either not pricked, pricked with a sterile needle, or pricked with DCV respectively (Martins, Faria, Nolte, Schlötterer, & Teixeira, 2014). Allele frequencies were compared using a CMH‐test and also using a binomial Generalised Linear Model (GLM). This study does not report levels of variation in allele changes between replicates but found that results were the same for the two statistical tests used (Martins et al., 2014). Other examples of E&R studies that use the CMH‐test to infer consistent allele frequency differences across replicates include response to novel laboratory environments (e.g. Huang, Wright, & Agrawal, 2014).

In all these cases, changes in allele frequency are taking place from a common base population in response to particular or directed selection. Studies also use these statistical methods to find SNPs associated with naturally divergent traits such as coat colour in domestic horse breeds (McCue et al., 2012), pigmentation variation in wild populations of *D. melanogaster* (Bastide et al., 2013), as well as loci influencing economically important traits (Ayllon et al., 2015). The same approach can also be used in case–control studies to find disease risk loci, which is conceptually identical to finding consistent allele frequency differences between two or more groups (e.g. Cichon et al., 2011; Mero et al., 2010).

While the above studies have yielded many promising results there is nevertheless an issue with the application of the CMH‐test which may result in numerous false positives. There is seldom any attempt reported at assessing whether candidate SNPs found conform to the assumptions of the CMH‐test, in particular the homogeneity of odds ratios. Such violations are likely to be common in many datasets and will produce false positives, which may be more frequent than true hits even after applying corrections for multiple testing. In fact, in a recent simulation study the CMH‐test was found to have very low precision in identifying SNPs under selection (Topa, Jónás, Kofler, Kosiol, & Honkela, 2015). Guides to E&R studies say that the CMH‐test performs better than some methods in other simulations (Kofler & Schlötterer, 2014) though these, and other, simulations do not seem to consider the special cases of pool‐seq designs (Baldwin‐Brown, Long, & Thornton, 2014; Kofler & Schlötterer, 2014). Meanwhile, other simulation studies have not considered a range of statistical approaches (Baldwin‐Brown et al., 2014; Kessner & Novembre, 2015) and consensus over best practices is lacking (Kessner & Novembre, 2015). Additionally, usually no attempt is made in studies to correct for the violations of independent counts although such corrections have been suggested in other contexts (e.g. Bergland, Behrman, O'Brien, Schmidt, & Petrov, 2014; Kolaczkowski et al., 2011; Machado et al., 2016).

### 1.3 Binomial GLMs, quasibinomial GLMs and linear models (LMs)

Another approach is to model allele frequencies in a GLM with binomial error distributions (binomial GLMs). This approach estimates the effects of a trait of interest, population of origin as well as their interaction on the allele or read count. This is similar to approaches that identify differential expression in RNA‐sequencing (RNA‐seq) experiments (Lund, Nettleton, McCarthy, & Smyth, 2012; McCarthy, Chen, & Smyth, 2012). Examples of binomial GLMs are less common in the literature to infer consistent allele associations with a stratum across population, although Martins et al. (2014) report using binomial GLMs to compare results with the CMH‐test. Binomial GLMs have been used to analyse allele frequencies in other contexts (e.g. Bergland et al., 2014; Jha et al., 2016; Kapun, Fabian, Goudet, & Flatt, 2016; Machado et al., 2016). A related statistical framework is the GLM with quasibinomial error distribution (quasibinomial GLM) that includes an extra parameter, φ, which can account for variation over and above that assumed by a binomial distribution (Crawley, 2013). This distribution has been useful in dealing with overdispersion in situations where frequency data are not well described by a binomial distribution. Finally, a General Linear Model (LM) is also possible where the allele frequencies are modelled as frequencies with the treatment group as a dependent variable. These approaches have the added benefit that they need not assume a specific pairing of an experimental treatment line with a ‘control’ or other line but a pairing can be added if there are good biological reasons to do so.

### 1.4 The G‐test

The G‐test is not commonly used in population genomics and is also based on the analysis of multi‐way contingency tables. The G‐test is similar to the Chi‐squared test but with more general application. The G‐test is based on the log‐likelihood ratio test, which is approximated at large sample sizes by the common Chi‐squared test (Sokal & Rohlf, 1969). The G‐test is less reliable when cell counts in the tables are 0 (Sokal & Rohlf, 1969, 1981) though continuity corrections where cell frequencies are low can make the test more robust (Sokal & Rohlf, 1969, 1981). The G‐test has not been applied to population genomic data.

The aim of this study is to assess, by simulation, the performance of different methods to identify consistent differences in allele frequencies between two treatment groups across biological replicates.

## 2 MATERIALS AND METHODS

### 2.1 Description of simulation protocol and parameter value choice

The behaviour of the G‐test, CMH‐test binomial and quasibinomial GLMs are explored using simulated datasets (see the supplementary material for the algorithm). 1,000,000 (of which 1% [10,000] were designated ‘true positives’, see below) independent SNP datasets are generated across *k* replicates of two treatment lines assuming a simple but realistic population genetic model that reflects a standard experimental evolution design. The neutral or ‘null’ case of an experimental evolution scenario can be described as an instantaneous fission model where *k* replicate subpopulations originate from a common ancestral population. Replicates are then split into two ‘treatment lines’ which evolve by drift for *t* generations leading to some differentiation (*F*_ST_ *> *0) from the ancestral population. We assume that the ancestral population is not under selection and is at mutation‐drift equilibrium. Thus, the ‘A’ allele frequency in the ancestral population (*p*_A_) is drawn from a beta distribution B(α*,* β) where:

$$\alpha \;=\;4N_{e}u$$

and,

$$\beta \;=\;4N_{e}v,$$

where *u* is the forward mutation rate and *v* is the backward mutation rate between the two states of a biallelic SNP respectively and *N*_e_ is the effective population size (Charlesworth & Charlesworth, 2008). The ‘A’ allele frequency within each ‘treatment line’ (*f*_A_) is generated as a sample from a truncated normal distribution bounded between 0 and 1 (Balding, 2003; Nicholson et al., 2002) with mean μ = *p*_A_ and variance σ^*2*^ *= F*_ST_ *p*_A_(1 − *p*_A_ *)*, where *F*_ST_ represents the amount of neutral divergence from the ancestral population (*F*_ST_) after *t* generations (Balding, 2003; Nicholson et al., 2002). No SNPs are allowed to become fixed for the same allele across all lines.

Because the entire population is rarely analysed in experiments a sample allele frequency at each locus of size *2N* = *n* alleles is drawn from each treatment line using the binomial distribution B(*n*,* f*_A_) so as to obtain the count (*x*) of the ‘A’ alleles in the sample. The count of ‘a’ alleles in the pool is then *n−x* and the frequency of ‘A’ in the pool (*f*_Apool_) is *x/n*. Finally, the counts among all the sequenced reads is given by another round of binomial sampling using *f*_Apool_ and total coverage (*CT*) by B(*CT*,* f*_Apool_). *CT* is either sampled or fixed and the allele counts (*C*_A_ and *C*_a_) reflect two rounds of binomial sampling (sampling from the treatment line and sampling from among the chromosomes with replacement). Data are thus generated by progressively filling the cells of contingency tables (shown algorithmically in the supplementary information). Each partial table represents a separate replicated pair of experimental evolution lines.

In pool‐seq data allele counts are commonly derived from raw read counts at each locus. This can lead to substantial variation in *CT* across genomic regions, and treatment lines due to differences in sequencing efficiency or random variation in coverage. Because a pool contains a fixed number of chromosomes (*n*) and *CT* can often exceed *n*,* C*_A_ and *C*_a_ are not independent draws, some chromosomes will be sampled more than once. This should be considered pseudoreplication (Feder, Petrov, & Bergland, 2012; Kolaczkowski et al., 2011). The double sampling nature of pool‐seq has been recognised and ways to deal with it have been proposed (e.g. Lynch, Bost, Wilson, Maruki, & Harrison, 2014). One way to ameliorate effects of this is to rescale counts to correspond to frequencies out of the known number of chromosomes in the sample or to a computed effective sample size (*n*_eff_) (Bergland et al., 2014; Feder et al., 2012; Kolaczkowski et al., 2011). Reviews of pool‐seq methods have offered ‘rule‐of‐thumb’ recommendations for sequencing coverage. Some recommend at least 50× but suggest up to 200× for reliable detection of low frequency alleles (Futschik & Shlötterer, 2010; Kofler & Schlötterer, 2014; Schlötterer et al., 2014). Others suggest between 1.4× and 4.1× per individual (Fracasetti, Griffin, & Willi, 2015). Here, simulations are run where *CT* is drawn from a negative binomial distribution with mean coverage 200 and a dispersion parameter of 2. After removing coverage values <10 this gives a realistic range of coverages from 18× to 859×. Alternatively, *CT* is fixed at 100, 200 or scaled using *n*_eff_. For the purposes of the *n*_eff_ correction, read depth is *CT* and sampled as above, the number of chromosomes/alleles in the pool (*n)* is 2*N* and *n*_eff_ is given by:

$$n_{\mathrm{ef}\;\;\;f}=\frac{\left(\left(n*CT\right)-1\right)}{\left(n+CT\right)}$$

according to Kolaczkowski et al. (2011) and Feder et al. (2012).

To parameterise the distributions in these simulations, it is necessary to take realistic values for the various population parameters. For mutation rate (*u* and *v*) values between 2 × 10^−9^ and 1 × 10^−8^ are common in e.g. *Heliconius melpomene* (Keightley et al., 2015) or *D. melanogaster* (Haag‐Liautard et al., 2007; Keightley, Ness, Halligan, & Haddrill, 2014) and estimates of *N*_e_ reported are on the order of 1,000,000–4,000,000 in these and other species (Charlesworth & Charlesworth, 2008; Jensen & Bachtrog, 2011; Keightley et al., 2014, 2015). Thus, the parameters of the beta distribution describing the allele frequencies in the base population are taken to be 4*N*_e_ *u =* *4N*_e_ *v* = 0.2. Several experimental evolution studies have recently been published (see Introduction). Many of these studies represent evolution over relatively few generations and few of them report standard population genetic divergence statistics. Nevertheless, when these data are available, fairly substantial *F*_ST_ estimates are typically reported. Kang, Aggarwal, Rashkovetsky, Korol, and Michalak (2016) report estimates of *F*_ST_ between 0.08 and 0.2 after ~50 generations of experimental evolution. Even after only three generations of evolution by drift, Santos et al. (2013) report differentiation of between 0.002 and 0.012. Some experimental evolution studies have been run for many more generations (~100 generations: Immonen, Snook, & Ritchie, 2014), in which case even higher estimates of *F*_ST_ are expected (~0.3–0.5). Neutral differentiation (*F*_ST_) will also depend on the population size. Here, we simulate data using values of 0.1, 0.2, or 0.3 for *F*_ST**,**_ which is probably conservative. Only results for *F*_ST_ = 0.2 are given below, results for *F*_ST_ = 0.1 and 0.3 are shown in the supplementary material. We assume a pool size (*N*) of 100 throughout which is on the same order of magnitude as other experimental evolution studies (e.g. Martins et al., 2014; Orozco‐terWengel et al., 2012a,2012b) and of recommended sample sizes (Schlötterer et al., 2014).

The primary aim of this study is to assess the False Positive Rates (FPRs) of different statistical tests. Under a null hypothesis a well‐behaved statistical test should produce a uniform distribution of *p*‐values ranging from 0 to 1 (Storey, 2002; Storey & Tibshirani, 2003). Thus, for a given cut‐off threshold α, the proportion of tests with a *p*‐value ≤ α should be α. This can be represented as a straight 1–1 line of the FPRs at different values of α against α on a log‐log plot. To evaluate the statistical tests in this study, the FPRs for α =** **0.0001, 0.0005, 0.001, 0.005, 0.01, 0.05, 0.1, and 0.5 is calculated for each test. The simulations are run to consider *k *=* *2, 3, 4, and 10 replicates. The CMH‐test, CMH‐test+Woolf‐test, binomial GLMs, quasibinomial GLMs, the G‐test, as described in Sokal and Rohlf (1969, 1981), and a LM are then applied to each simulated SNP. Because the allele frequencies produced in these simulations are random draws and the population genetic model applied is a neutral one, the simulations represent a null or ‘neutral’ scenario and most simulated SNPs are expected to show no consistent difference across the *k* samples.

While the main aim of this study is to evaluate the FPRs of these statistical tests, the ability of tests to identify true positives is also of interest. Thus, 1% of the 1 million SNPs (10,000 SNPs) are designated ‘true positives’. For each true positive the SNP frequencies are simulated as above with the exception that one treatment is given a consistent allele frequency increase in 0.2 on top of whatever change is generated by drift. The ‘True Positive Rate’ (TPR) can then be roughly assessed by estimating the proportion of all true positives recovered among the bottom 1% SNPs of the *p*‐value distribution.

### 2.2 Implementation of the CMH‐test

CMH‐tests are performed using the R function mantelhaen.test() from the ‘stats’ package. This same function is used in the popular software package popoolation2 (Kofler et al., 2011). Heterogeneity was tested using a Woolf‐test (Woolf, 1955) from the same r package. Counts of zero are tolerated by the CMH‐test but not by the Woolf‐test where a common procedure is to add one to each zero count cell. Here, one is added to all cells if there are any empty cells for both the Woolf‐test and the CMH‐test. In the CMH‐test framework, a consistent result should be one that shows a common odds ratio significantly greater than one as well as a non‐significant test of heterogeneity in odds ratios.

### 2.3 Implementation of binomial GLMs, quasibinomial GLMs and LMs

GLMs are run in the standard R glm() function, from the ‘stats’ package. Two model structures are tested for binomial GLMs:

(1) y=treatment+replicate+treatment:replicate+e

and,

(2) y=treatment+e

where *y* gives the counts of ‘A’ and ‘a’ alleles, treatment, replicate, and treatment:replicate are the treatment, replicate, and interaction effects respectively. *e* is a binomially distributed error term. A consistently associated SNP is one where there is both no evidence for a two‐way interaction between treatment line and replicate on allele frequency (L × R interaction) and an overall significant effect of treatment line (L) on allele frequency, this is tested by model structure (1). Model structure (2) simply tests whether there is an overall effect of treatment. Inconsistent allele frequency differences should increase variance in one treatment and give non‐significant treatment effects. Under model structure (1) *p*‐values for the treatment and interaction effects are obtained from likelihood ratio tests. For model structure (2) *p*‐values are from *t*‐tests. Counts of zero are tolerated by the GLM but can lead to other problems due to fitted values from the link function being undefined. To counter this, a common procedure is to add a count of one to each allele count if any zero counts are encountered, which we adopt here. Quasibinomial GLMs are also fitted with the glm() function (family = ‘quasibinomial’). Only the model structure (2), see above, is tested because there are not enough residual degrees of freedom to test for interaction effects. Interaction effects are estimated for binomial GLMs because dispersion is assumed to be 1. However, these estimates should be treated with a degree of caution. For quasibinomial GLMs, *e* is a quasibinomially distributed error term and *p*‐values for the treatment effects are obtained from *t*‐tests. Finally, a general Linear Model (LM) is implemented with model structure (2) in the function lm(). In the LM, *e* is the Gaussian error term. *p*‐values for the treatment effects are obtained by *t* tests.

### 2.4 Implementation of the G‐test

G‐tests are performed as described in (Sokal & Rohlf, 1969) using a custom written R function. Here, a SNP allele that occurs at consistently different frequencies between lines across populations is one which shows an overall association between allele and line (L x A) as well as a non‐significant line by population by allele count interaction (L × A × P interaction). Again, one is added to all cells if any cells are empty.

All simulations and analyses were performed in the r programming language (R Development Core Team, 2014). All code, including custom written functions are available at: https://github.com/RAWWiberg/ER_PoolSeq_Simulations. Data presented below are archived in the Dryad repository: http://dx.doi.org/10.5061/dryad.mn0tv.

### 2.5 Re‐analysis of a dataset

Data from the E&R study on adaptation to novel temperature environments in *D. melanogaster* is re‐analysed (Orozco‐terWengel et al., 2012a,2012b). Raw data files, as generated by the popoolation2 package, are available from Dryad (Orozco‐terWengel et al., 2012a, 2012b; http://dx.doi.org/10.5061/dryad.60k68.2). These data are re‐analysed using quasibinomial GLMs as above. The original data analysis is described in Orozco‐terWengel et al. (2012a,2012b), and also re‐analysed in Topa et al. (2015) and Iranmehr, Akbari, Shlötterer, and Bafna (2016). Here, we compare the results from the original study and re‐analyse the raw data with some modifications. The full dataset contains 1,547,837 SNPs from six pools of 500 individuals each. We consider only truly biallelic SNPs, as in Topa et al. (2015). The minimum and maximum coverage thresholds remain as in Orozco‐terWengel et al. (2012a,2012b) (min‐count = 10, min‐cov = 10, max‐cov = 500). Analyses are performed on the raw allele counts and counts re‐scaled to be out of either 1,000 (to match the number of independent chromosomes in the pool), 100 or *n*_eff_. In total, 1,370,371 SNPs are analysed. The base (B) and 37^th^ generation (F37) from the experiment are compared across three replicated experimental evolution lines in order to identify consistent allele frequency differences across the three replicates.

## 3 RESULTS

### 3.1 Simulated dataset

The distributions of the mean allele frequency difference between the lines and the standard deviation (*SD*) of these differences are shown in Figures S1‐S3. The *SD* can be viewed as a measure of how consistent the difference between the two treatment groups is. The *SD* is inexact since its calculation requires a pairing of treatment lines while some statistical tests do not assume a pairing and in many experimental designs no meaningful pairing exists. There is no systematic relationship between the mean allele frequency difference and the *SD* of allele frequency differences indicating that these are varying freely in the simulations.

### 3.2 False positive rates

**Figure 1:**
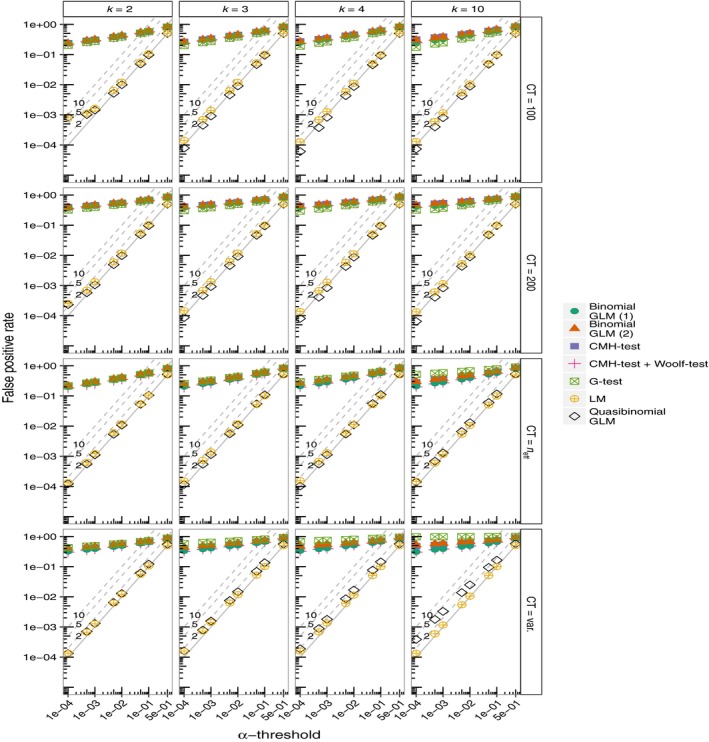
The False Positive Rates (FPRs) at different levels of α for each simulation. Simulations are run for *k* = 2, 3, 4, and 10 replicated treatment lines with a neutral *F*_ST_ of 0.2. Allele counts are allowed to vary freely (CT = var.), are fixed at 100 or 200 (CT = 100, CT = 200), or are scaled to neff (CT = *n*_eff_). Binomial GLMs are run with model structure (1) or (2) (see Methods). The diagonal lines labelled ‘2’, ‘5’, and ‘10’ represent 2, 5, and 10‐fold inflations of *p*‐values respectively. Pool size (*N*) is 100 diploid individuals, thus the number of independent chromosomes is 2*N* = 200

There is substantial variation in the FPRs of each of these tests (Figure 1). It is clear that the FPRs for the CMH‐test are seriously overinflated even at very stringent values of α. FPRs are greater where allele frequencies are given by the raw allele counts which are allowed to vary (Figure 1). Although Figure 1 suggests that the differences among the panels are small, on the log‐log scale they are in fact substantial. Nevertheless, FPRs are high in all cases. This is also seen in the histograms of the *p*‐values (Figure S4). The FPRs are also highly inflated even when the Woolf‐test is used in an attempt to identify SNPs where the odds ratios are not homogenous across the partial tables (Figure 1). Similarly, the FPRs are high for the G‐test, as well as binomial GLMs. In contrast, GLMs with a quasibinomial error distribution and the regular LM show FPRs that are more appropriate. Both approaches (the LM and quasibinomial GLMs) produce FPR lines that lie very close to the expected 1–1 line (Figure 1). This is also clear from the histograms of the *p*‐values (Figure S5). The largest inflations of FPRs are again seen in simulations where the allele counts are allowed to vary and are very low in the simulations where allele counts are fixed at 100, 200 or scaled to the effective sample size (*n*_eff_) (Figure 1). In terms of the FPRs it is clear that the quasibinomial GLMs and the LMs perform best. Patterns of FPRs are not affected by assuming different values of *F*_ST_ (Figures S6 and S7).

### 3.3 True positive rates

**Figure 2:**
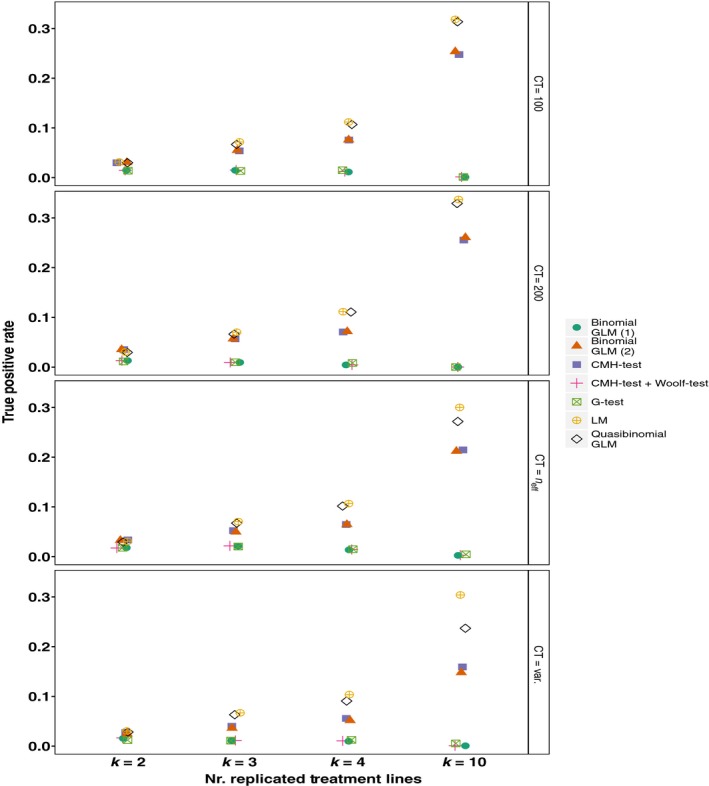
The True Positive Rates (TPRs). The TPR is calculated as the proportion of 10,000 true positives that are recovered among the top 1% of SNPs. Simulations are run for *k* = 2, 3, 4, and 10 replicated treatment lines with a neutral *F*_ST_ of 0.2. Allele counts are allowed to vary freely (CT = var.), are fixed at 100 or 200 (CT = 100, CT = 200), or are scaled to neff (CT = *n*_eff_). Binomial GLMs are run with model structure (1) or (2) (see Methods). Pool size (*N*) is 100 diploid individuals, thus the number of independent chromosomes is 2*N* = 200. Points have been “jittered” horizontally to avoid overlap

These simulations also implemented a simple method for assessing the power of the different tests. The TPR is calculated as the proportion of all true positives that were seeded in the simulations that are recovered among the SNPs below the 1^st^ percentile of the *p*‐value distributions (hereafter the ‘top 1% of SNPs’). In general the CMH‐test seems to perform quite well recovering between ~15% and 29% of true positives. However, quasibinomial GLMs and LMs perform better, recovering ~30% of true positives among the top 1% of SNPs (Figure 2). The remaining statistical tests (binomial GLMs and G‐tests) perform rather poorly recovering less than 5% of true positives. While there are some differences in the TPRs as the allele counts are allowed to vary or kept fixed, the TPR is primarily influenced by the number of replicates (Figure 2). Precise TPRs will vary with how large the average difference between treatment lines due to selection is in comparison to neutral differentiation among the treatment lines. In this simulation the value added consistently to one treatment as a difference due to selection was 0.2. Thus, these values should be taken as a guide, though the distribution of TPRs from multiple simulations with the same parameters is narrow, especially for simulations of 1,000,000 SNPs (Figure S8). Patterns of TPRs are affected by assuming different values of *F*_ST_ while keeping the difference between treatments at true positives the same, but the relative performance of the tests remains the same (Figures S9 and S10).

### 3.4 Re‐analysis of dataset

**Figure 3:**
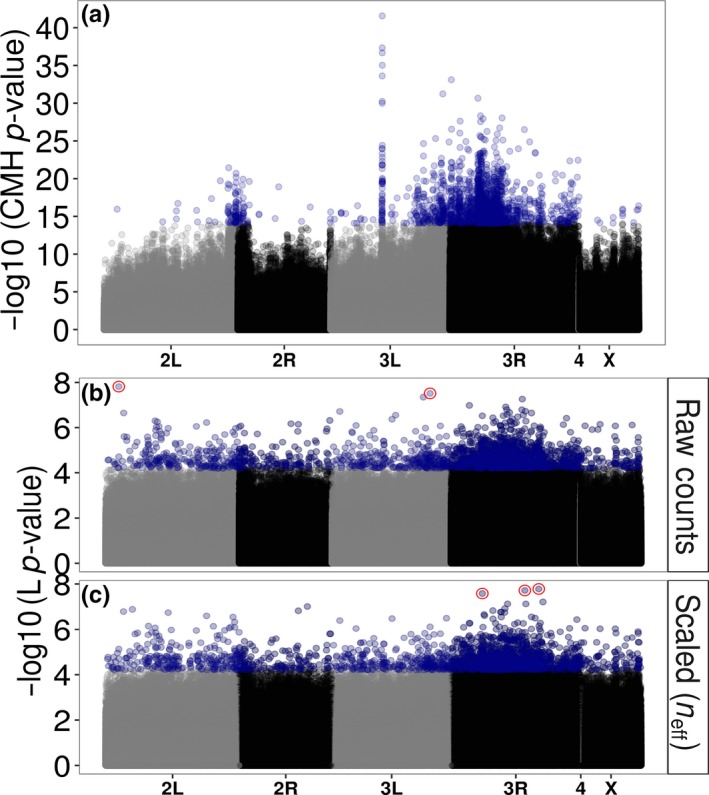
(a) Manhattan plot of the original CMH‐test results from Orozco‐terWengel et al. (2012a,2012b). Blue points are the top 2,000 SNPs identified by the CMH‐test. Also shown are manhattan plots of the re‐analysis of the Orozco‐terWengel et al. (2012a,2012b) data using Quasibinomial GLMs on (b) the raw counts as well as (c) scaling counts to neff. Shown are the −log10 (*p*‐values) from the main treatment line (L) effect. Blue points are the top 2,000 SNPs. Red points in (b) and (c) are SNPs that pass genome‐wide Bonferroni correction. Note the differences in scale on the *y*‐axis across the panels

The analysis of allele frequencies from raw counts produces somewhat similar results to the original analysis (Orozco‐terWengel et al., 2012a,2012b) (Figure 3). Spurious false positives due to excessive coverage near chorion gene clusters on chromosome 3L (Orozco‐terWengel et al., 2012a,2012b) are no longer apparent (Figure 3b,c). However, scaling counts to match the large number of chromosomes in the pools (to be counts out of either 100 or 1,000) produces unusual looking Manhattan plots (Figure S11), likely because it creates artificially high confidence in the measurements within the quasibinomial GLM resulting in inflated −log10 (*p*‐values). A random sample of 100 of the SNPs that are significant after Bonferroni correction suggest that these high scoring SNPs still show patterns that researchers would want to identify, i.e. they show a consistent difference between the two time points across replicates (Figures S12 and S13). Using raw allele counts or scaling counts to correspond to *n*_eff_ does not produce this inflation (Figure 3b,c).

Because quasibinomial GLMs produce the expected uniform distribution of *p*‐values under the null hypothesis (Figure 1), it is possible to apply standard corrections for multiple testing. The number of SNPs that achieve genome‐wide significance using q‐values (Storey, Bass, Dabney, & Robinson, 2015; Storey & Tibshirani, 2003), Benjamini–Hochberg (B–H) (Benjamini and Hochberg 1995), or Bonferroni correction are shown in Table 2. It is apparent that raw counts and counts scaled to *n*_eff_ are more conservative estimates at least for the Bonferroni correction. Methods that control the FDR (Q‐values and B–H correction) are far more liberal and produce more ‘significant’ SNPs (Table 2).

**Table 2:** The number of SNPs (single nucleotide polymorphisms) that pass multiple test correction in the re‐analysed datasets. For Bonferroni correction the α threshhold 0.05 was divided by the total number of tests (SNPs tested) to get the genome‐wide multiple test correction threshold. For *Q*‐values and Benjamini–Hochberg (B–H) correction, a False Discovery Rate (FDR) threshhold of 0.05 was used. Bonferroni corrections carried out manually, B–H corrections followed the procedure in Benjamini and Hochberg (1995), and *q*‐values were calculated using the “qvalues” package in r (Storey et al., 2015)

Re‐analysis	Bonferroni	*Q*‐values	B–H
Raw counts	2	67,702	3,961
Counts scaled to *n*_eff_	3	67,505	4,571
Counts scaled to 100	456	61,022	15,053
Counts scaled to 1,000	33	13,013	2,532

## 4 DISCUSSION

With the increasing popularity of pooled‐sequencing methods to study population genomics and E&R studies, the importance of determining best practice statistical methods for allele frequency estimation and the identification of consistent allele frequency differences is crucial. User‐friendly software packages remove the need for complicated scripting but make statistical tests less transparent. This study highlights problems with the way in which the popular CMH‐test is applied and proposes some alternatives.

The CMH‐test produces a large number of significant test results under the null hypothesis and as such has very high FPRs even at relatively high α thresholds. This seems to be because it confounds heterogeneity and a main effect. Indeed, the potential for this is noted in much of the original literature describing this test (Agresti, 1996; Landis et al., 1978). Similarly, many other statistical tests assessed here have FPRs that are unacceptably high (G‐tests and binomial GLMs). However, LMs and quasibinomial GLMs perform well under the null hypothesis, producing uniform *p*‐value distributions and the characteristic 1–1 relationship, on the log‐log scale, between the FPR and different thresholds of α.

True Positive Rates, the ability to identify SNPs that are in fact under selection (true positives), varies across the tests and simulations. Quasibinomial GLMs and LMs perform best, recovering more true positives than other statistical tests. As expected, keeping the difference applied at true positives the same while reducing the neutral differentiation (*F*_ST_) increases the TPR. In addition, as in other simulation studies (Baldwin‐Brown et al., 2014; Kessner & Novembre, 2015; Kofler & Schlötterer, 2014), there is a strong relationship with the number of replicated treatment lines in the experiment regardless of the statistical test (and our simulations suggest that most current studies are underpowered here).

In addition to low FPRs and high TPRs, quasibinomial GLMs have other attractive features. First, properly behaved *p*‐values allow controlling FDRs e.g. by *q*‐values, Bonferroni or Benjamini‐Hochberg correction (Storey & Tibshirani, 2003). This is preferable to relying on arbitrary cut‐offs of e.g. ‘the top 1%’ or the ‘top 1,000’ SNPs. Another attractive feature of quasibinomial GLMs is that there is no need to arbitrarily pair experimental treatments but the option exists if it makes biological sense and if the data allow it. GLMs also allow for more complicated nested experimental designs and interactions. Mixed models could also incorporate random effects.

The simulations in this study highlight an additional problem. When pools of individuals are sequenced, the coverage can vary substantially between pools or between genomic regions. This translates to differences in the total count for a SNP position. Our results indicate that variation in these counts between loci affects the performance of some statistical tests. A simple solution is to rescale all allele counts to represent either a proportion out of a fixed number that reflects how many alleles are in the pool (i.e. how many chromosomes are being sequenced) or to the effective sample size *n*_eff_ (Feder et al., 2012; Kolaczkowski et al., 2011). Results from the re‐analysis of the Orozco‐terWengel et al. (2012a,2012b) dataset suggest that *n*_eff_ is preferable.

Re‐analysis of the Orozco‐terWengel et al. (2012a,2012b) dataset also showed improvements in the consistency of the allele frequency difference between treatment lines across replicates in the top SNPs identified. The results were qualitatively similar to previously published analyses with peaks and troughs in the same genomic regions (Figure 3) although very few SNPs pass Bonferroni correction for multiple testing (Table 2). Furthermore, the large peak on chromosome 3 that is attributed to artefacts of higher coverage in Orozco‐terWengel et al. (2012a,2012b) is no longer visible (Figure 3).

In summary, the results presented here indicate that reliable identification of SNP alleles that occur at consistently different frequencies in different treatment lines across biological replicates of natural populations or experimental evolution lines requires two things. First, an appropriate statistical test needs to be chosen that does not confuse heterogeneity for a main effect. Two such tests, quasibinomial GLMs and linear models, are available and produce appropriate FPRs and TPRs, and also have other attractive properties. Second, variation in coverage across SNPs and replicates affects results in some circumstances. However, standardising coverage should be done with care because if the counts are too high this will create an artificially high level of confidence in overall effects, resulting in very low (effectively zero) *p*‐values. The effective sample size procedure seems useful and is well grounded in theory (Feder et al., 2012; Kolaczkowski et al., 2011). Finally, power (TPRs) seems to be related primarily to the number of replicates per treatment within the experiment although the strength of selection in comparison to the neutral divergence (*F*_ST_) also plays a role. It is important to appreciate that several studies have used the CMH‐test and successfully uncovered biologically meaningful loci confirmed by additional functional analyses (e.g. Martins et al., 2014), Thus it is clear that conclusions drawn in such studies are still valid despite these potential issues with the CMH‐test.

Throughout this study we have followed the convention that the more important loci to identify are those which diverge consistently across replicate treatment lines. It is commonly argued that such loci are those most likely to represent responses to divergent selection, because inconsistent divergence may be due to drift. However, it is probably worth noting that evolutionary responses can often be opportunistic. Different SNPs segregating within genes or regulatory regions may provide alternate responses to similar selection pressures or some forms of selection (e.g. parasite–host co‐evolution or sexual selection) may be particularly likely to cause inconsistent responses. Hence, not all loci showing inconsistent responses in real datasets will be false positives.

## AUTHORS’ CONTRIBUTIONS

R.A.W.W., M.B.M., O.E.G. and M.G.R. conceived the ideas and designed the simulation methodology; R.A.W.W. performed the analyses and simulations. R.A.W.W. led the writing of the manuscript. All authors contributed critically to the drafts and gave final approval for publication.
