# Supplementary material for: Identifying consistent allele frequency differences in studies of stratified populations
Source: Methods Ecol Evol. 2017 Jun 15;8(12):1899–909. doi: 10.1111/2041-210X.12810 (PMC5726381; doi:10.1111/2041-210X.12810)
Supplement: Supplementary file 1 [file MEE3-8-1899-s001.doc]

**Simulation Algorithm:** A pseudocode description of the algorithm for building the null hypothesis contingency table data used in these analyses. See main text for the values of each parameter used. Indentation reflects different hierarchies of loops and computational steps are delineated by a “-”. Variables are highlighted in **bold**. Sequences of “:” delineate levels of the pseudo “for-loop”

For each SNP:

: Sample the frequency of the “A” allele (***p_A_***) in the base population.

B(a=0.2,b=0.2)

: For each replicate (***k***)::

:: For each line (***l***):::

::: Sample the frequency of the “A” allele in the line (***f_A_***) from a truncated normal distribution.

tN(mean=***p_A_***,sd=sqrt(***F_ST_***(***p_A_***(1-***p_A_***)),min=0,max=1)

::: Sample the “A” allele counts in the sample (***x***) from a binomial distribution.

bin(n=1,size=***n***,p=***f_A_***)

::: The frequency of the “A” allele in the pool (***f_Apl_***) is then ***x****/****n*** = ***f_Apl_.***

::: Sample or fix the row counts (***CT***). If ***CT*** is sampled it is from:

nbin(mu=**mcov**,d=2)

::: Sample the pool “A” allele count (***C_A_***) from:

bin(n=1,size=***CT***,p=***f_Apl_***)

::: Compute the pool “a” allele count (***C_a_****=* ***CT****-****C_A_***)*.*

: Run statistical tests for the SNP and save the results.

**Supplementary Figures and Legends**

**
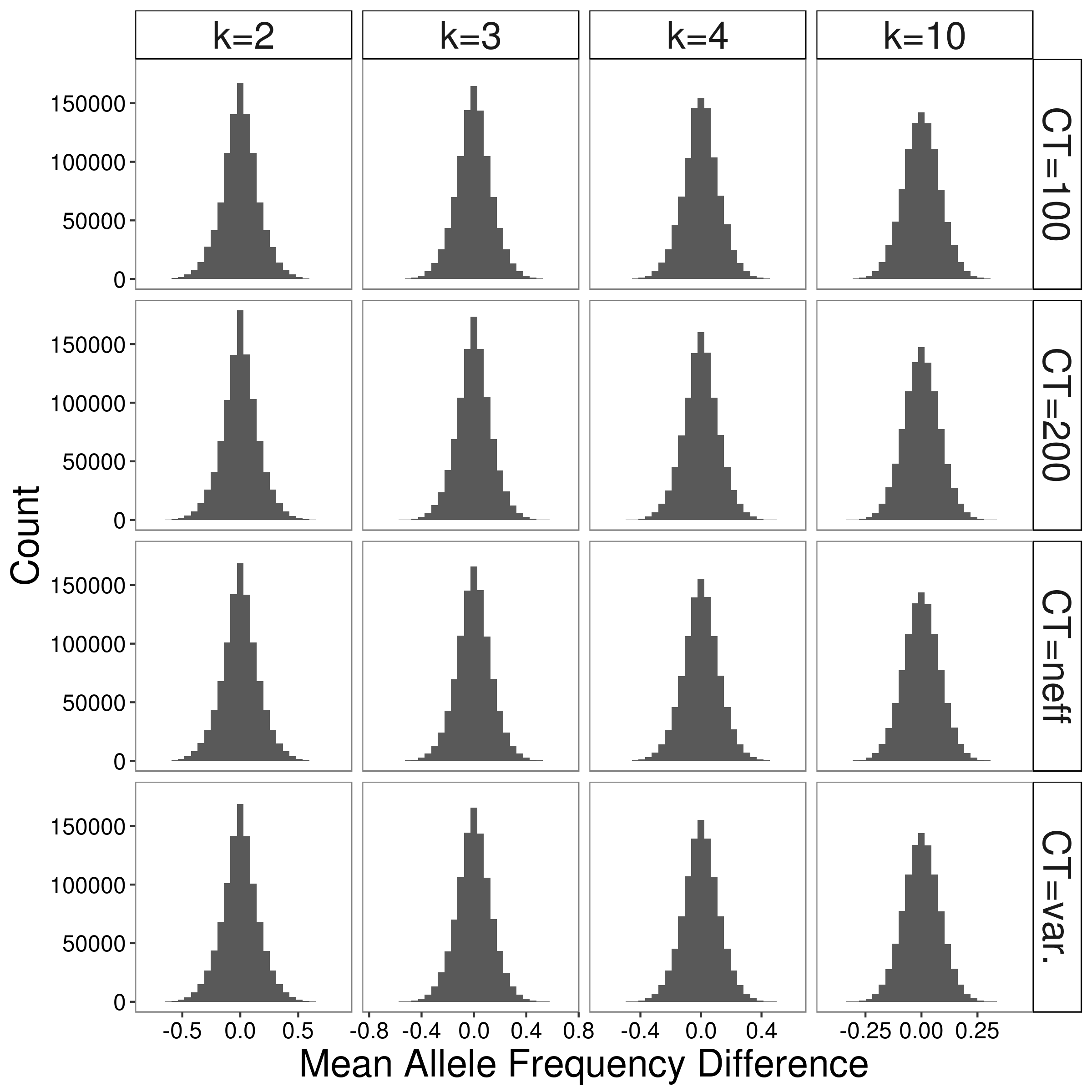
**

**Figure S1.** Distribution of mean allele frequency differences between treatment lines across replicates for each of the simulations. Data are shown for simulations that consider *k =* 2, 3, 4, and 10 replicates. “CT=var.” - row totals in the 2-way tables are sampled from a negative binomial distribution, “CT = 100” - row totals in each of the 2-way tables are fixed at 100, “CT = 200” - row totals in each of the 2-way tables are fixed at 200, “CT = neff” - row totals in the partial tables are scaled to the effective sample size. Only the “neutral” SNPs are shown.


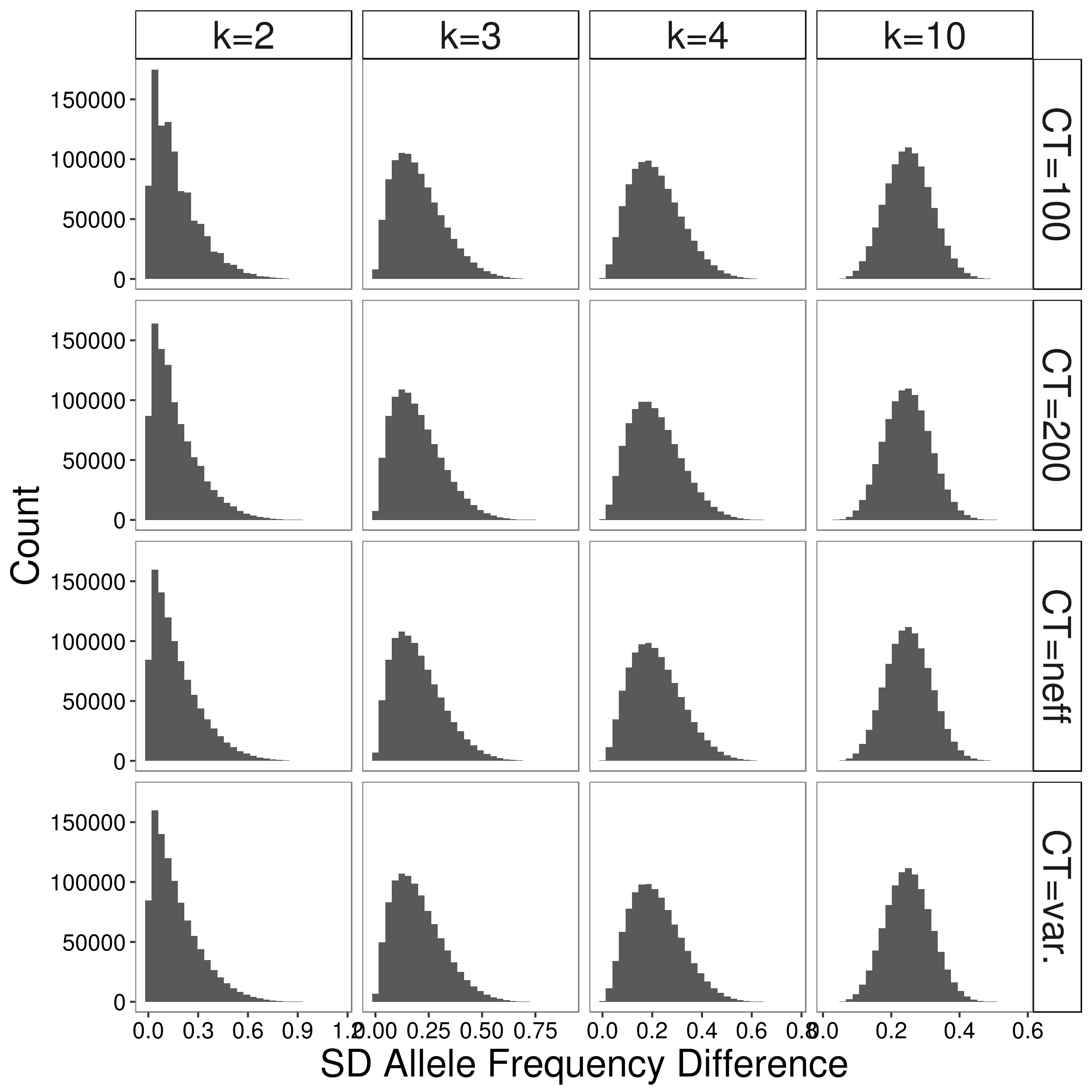


**Figure S2.** Distribution of the standard deviation (SD) of allele frequency differences between treatment lines across replicates for each simulation. Labels are as in Figure S1.


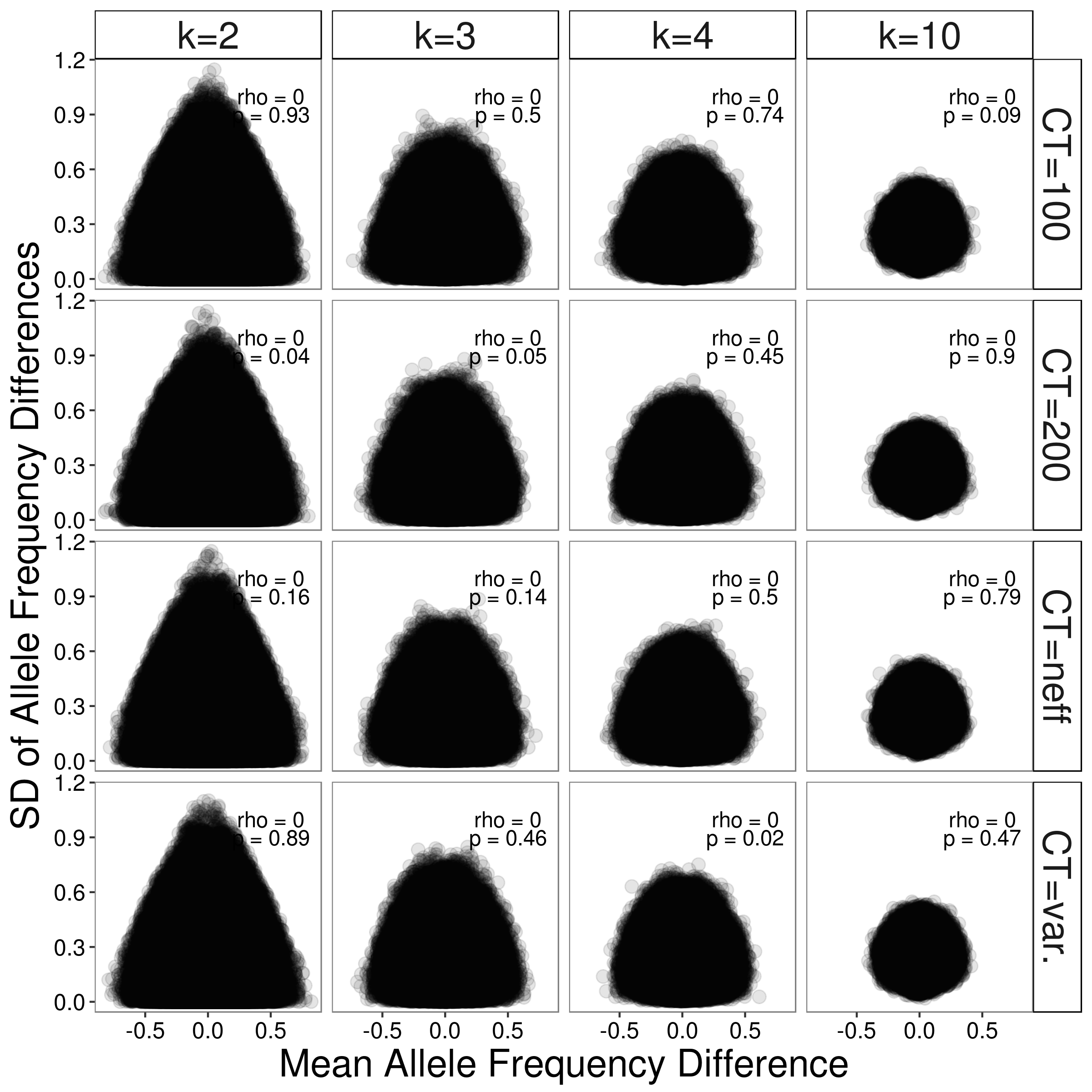


**Figure S3.** Relationship between the mean difference in allele frequencies between treatment lines, across replicates and the standard deviation (SD) of allele frequency differences. The data shown are from 1,000,000 simulations. Labels are as in Figure S1. Inset text gives the p-values and correlation coefficients (rho) for Spearman Rank correlation tests between the *x* and *y* variables.


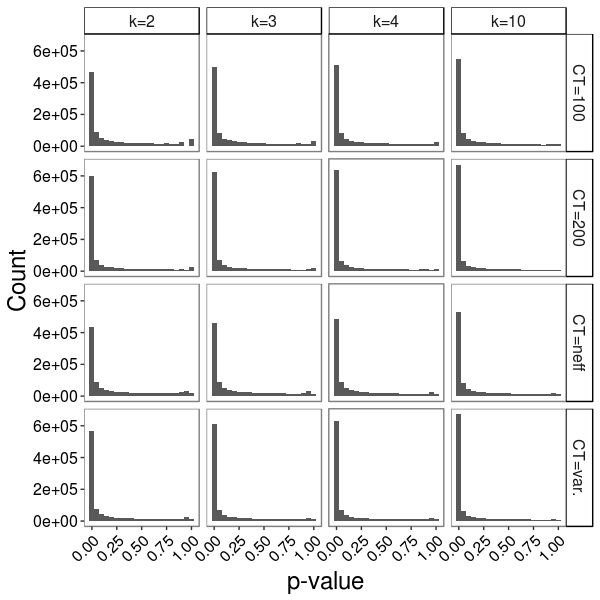


**Figure S4.** Histograms of p-values under the null hypothesis for a CMH-test under the different simulations. Labels are as in Figure S1.


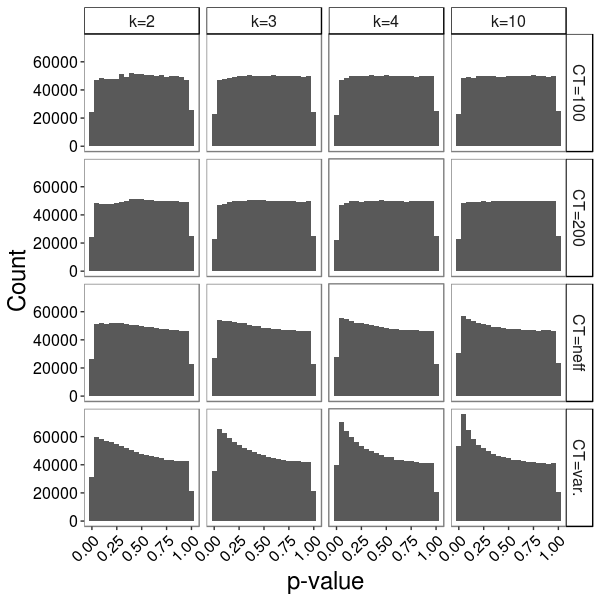


**Figure S5.** The histograms of p-values under the null hypothesis for a Quasibinomial GLM under the different simulations. Labels are as in Figure S1.


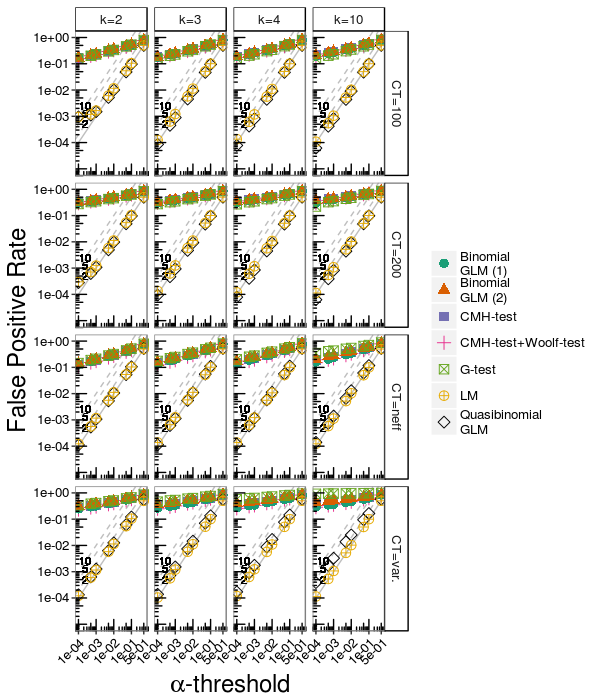


**Figure S6.** FPRs for simulations where *F*_ST_ = 0.1. Figure labels are as in Figure 1 of the main text.

**
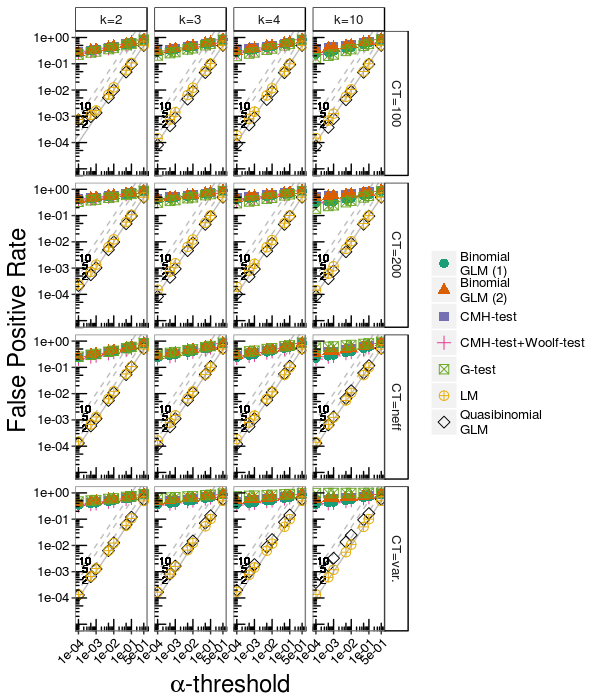
**

**Figure S7.** FPRs for simulations where *F*_ST_ = 0.3. Figure labels are as in Figure 1 of the main text.


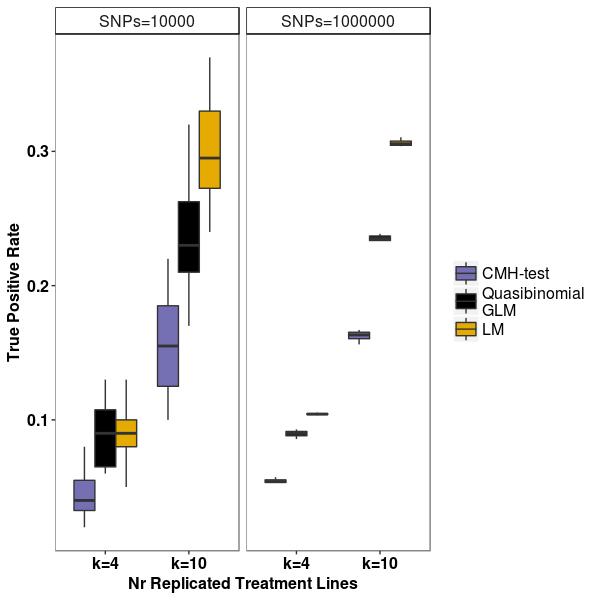


**Figure S8.** The consistency and scaling of the True Positive Rate (TPR) across simulations. Results are shown for 10 repeated simulations of 10,000 SNPs at *k* = 4 or 10 (left panel), or 4 repeated simulations of 1,000,000 SNPs at *k* = 4 or 10 (right panel). In all simulations allele counts are scaled to be out of 100, neutral divergence (*F*_ST_) is set to 0.2, and the a difference between treatment lines applied to simulate an average difference due to selection is 0.2, as in the main results (Figure 2). Results for the CMH-test, Quasibinomial GLMs and LMs are shown.


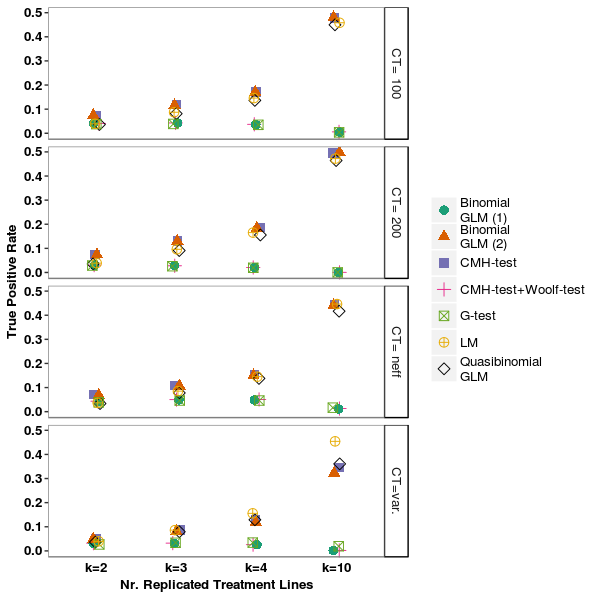


**Figure S9.** TPRs for simulations where *F*_ST_ = 0.1. Figure labels are as in Figure 2 of the main text.

**
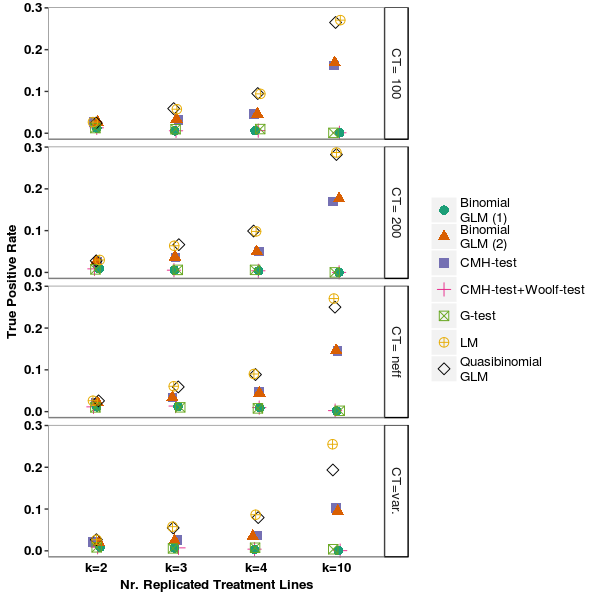
**

**Figure S10.** TPRs for simulations where *F*_ST_ = 0.3. Figure labels are as in Figure 2 of the main text.

**
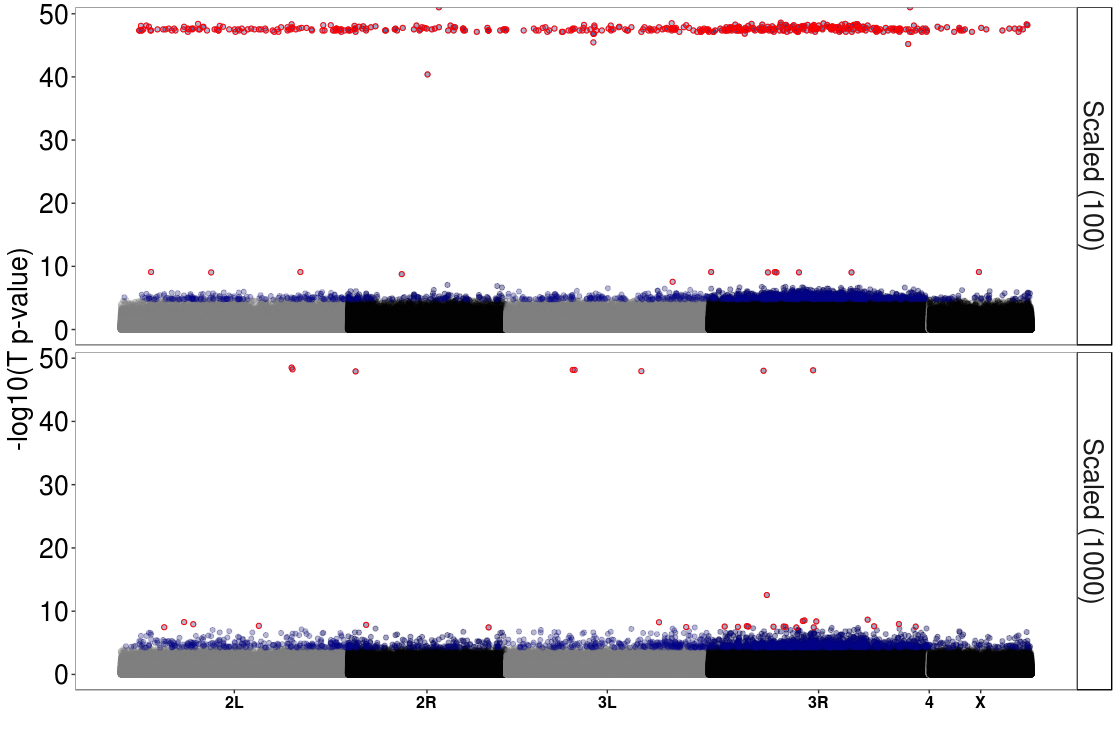
Figure S11.** Manhattan plots for the re-analysis of the Orozco-terWengel et al., (2012) dataset where allele counts have been scaled to be out of 100 (top panel) or 1,000 (lower panel). Blue points show the top 2,000 SNPs. The points circled in red are SNPs that pass the genome-wide Bonferroni correction threshold.

**
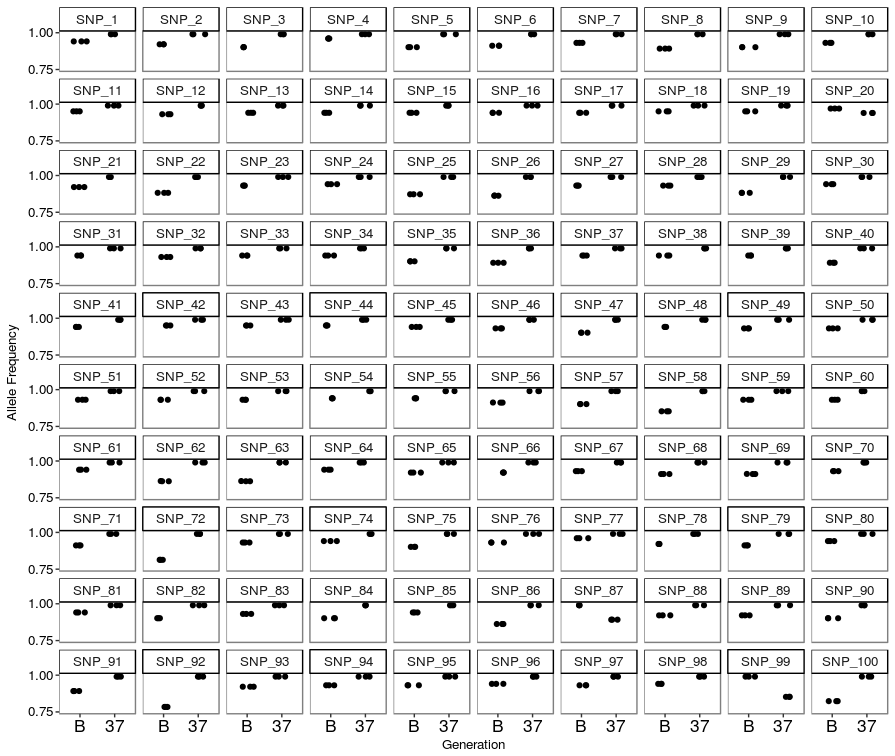
Figure S12.** A random sample of 100 SNPs from the SNPs that pass genomewide Bonferroni significance threshold in the re-analysis of the Orozco-terWengel et al., (2012) dataset where allele counts have been scaled to be out of 100 (Figure S7). Points have been horizontally “jittered” to prevent overlap.

**
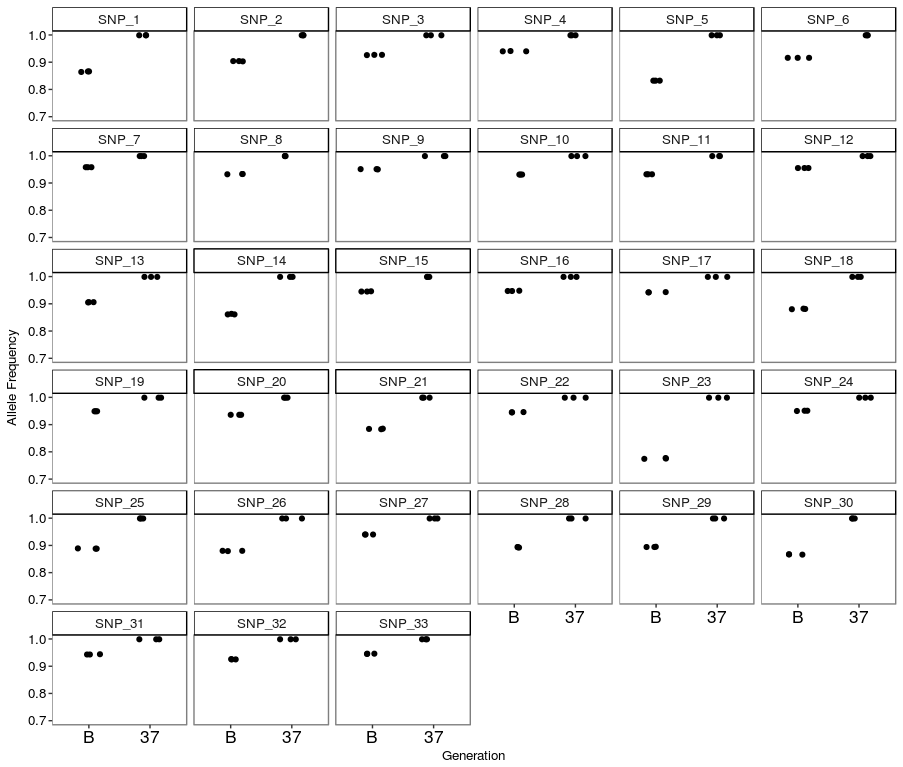
Figure S13.** The 33 SNPs that pass genomewide Bonferroni significance threshold in the re-analysis of the Orozco-terWengel et al., (2012) dataset where allele counts have been scaled to be out of 1,000 (Figure S11). Points have been horizontally “jittered” to prevent overlap.
